# Supplementary material for: Coordination of actin plus-end dynamics by IQGAP1, formin, and capping protein
Source: J Cell Biol. 2024 May 24;223(9):e202305065. doi: 10.1083/jcb.202305065 (PMC11117073; doi:10.1083/jcb.202305065)

Source Data F1B. Coomassie gel of 6xHis purification

Figure Panel

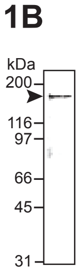

Labeled Source

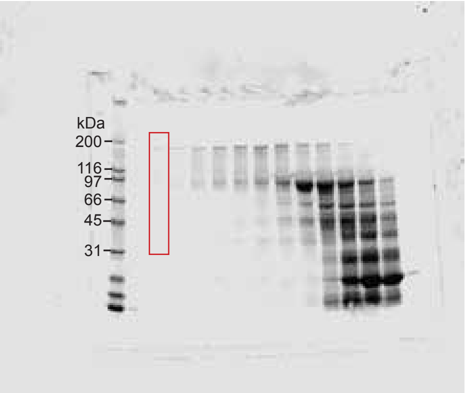

Unlabeled Source

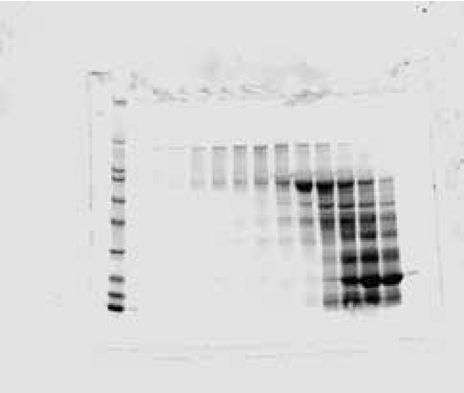

Supplement: SourceData F1 — is the source file for Fig. 1. [file JCB_202305065_SourceDataF1.pdf]
