# Supplementary figures and images for: Coordination of actin plus-end dynamics by IQGAP1, formin, and capping protein
Source: J Cell Biol. 2024 May 24;223(9):e202305065. doi: 10.1083/jcb.202305065 (PMC11117073; doi:10.1083/jcb.202305065)

Figure Panel

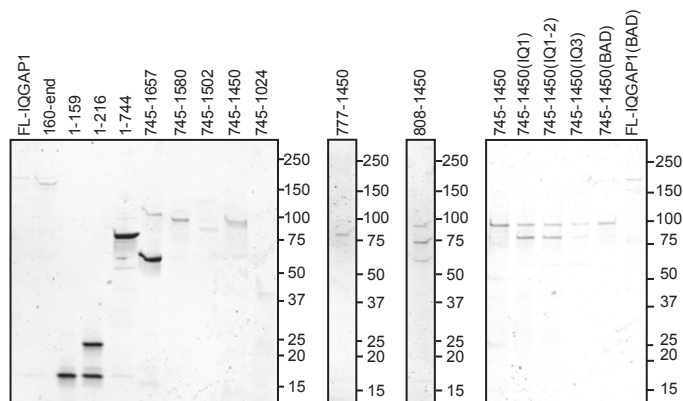

### Labeled Source

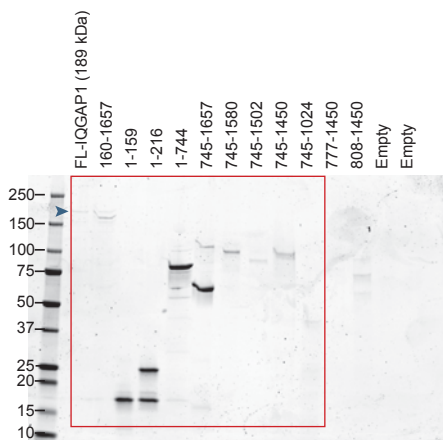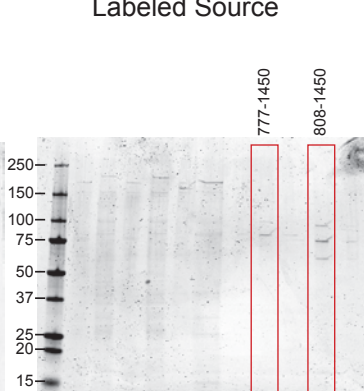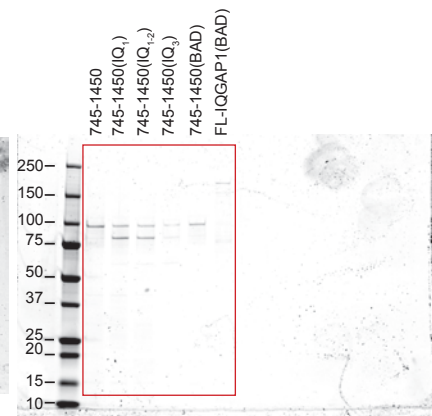

Unlabeled Source

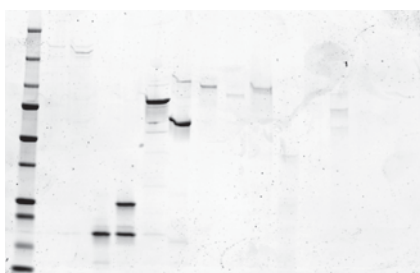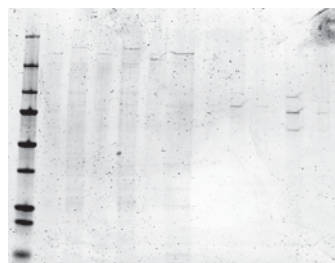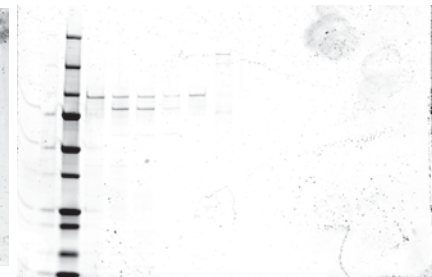

Supplement: SourceData FS1 — is the source file for Fig. S1. [file JCB_202305065_SourceDataFS1.pdf]

Figure Panel

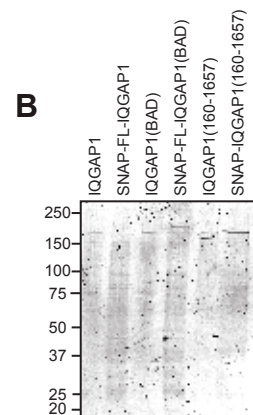

Labeled Source

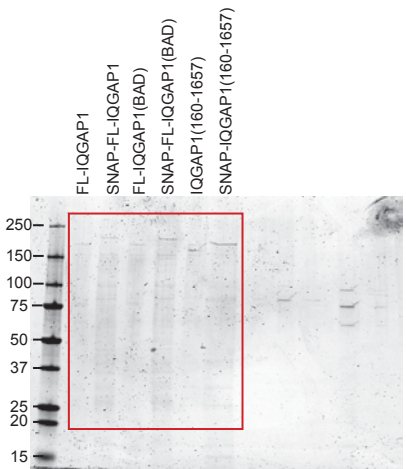

Unlabeled Source

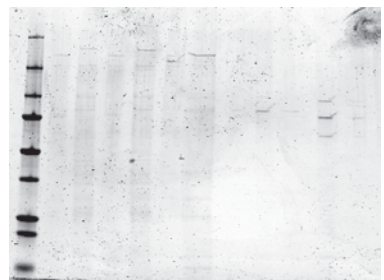

Supplement: SourceData FS2 — is the source file for Fig. S2. [file JCB_202305065_SourceDataFS2.pdf]
