# Supplementary material for: Coordination of actin plus-end dynamics by IQGAP1, formin, and capping protein
Source: J Cell Biol. 2024 May 24;223(9):e202305065. doi: 10.1083/jcb.202305065 (PMC11117073; doi:10.1083/jcb.202305065)

# Source Data FS4A. Western blots of NIH-3T3 cell extracts

## Figure Panel

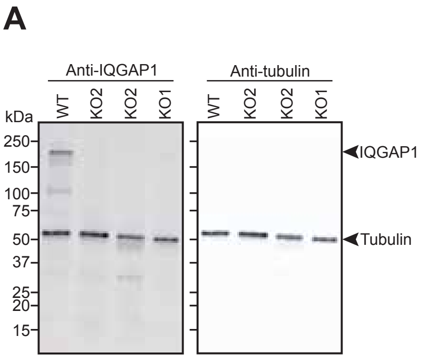


## Labeled Source

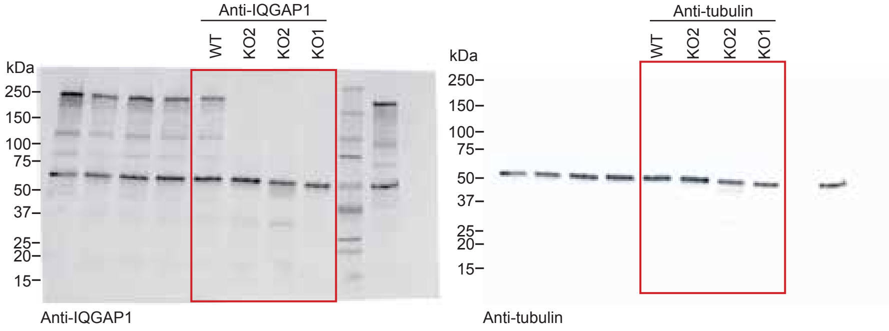


## Unlabeled Source

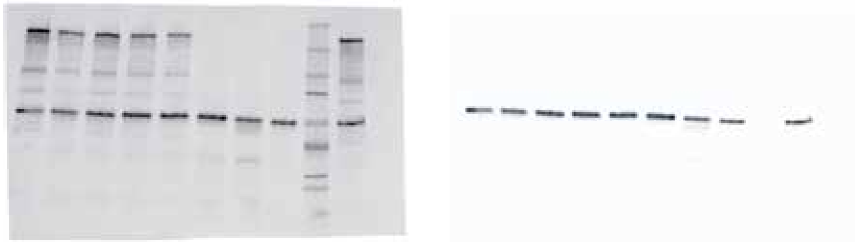

# Source Data FS4B. Western blots and Ponceau of NIH-3T3 cell extracts

## Figure Panel

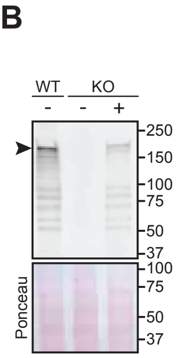


## Labeled Source

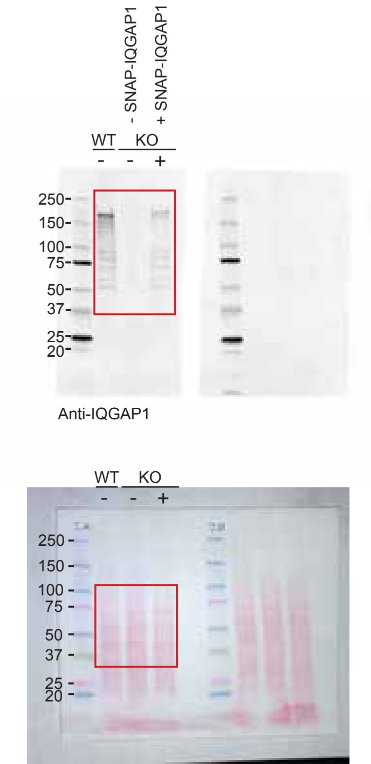


## Unlabeled Source

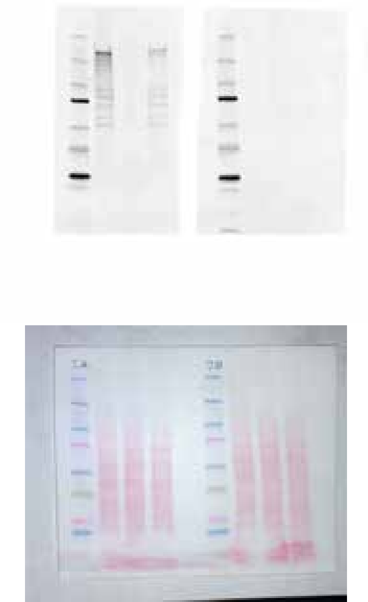

Supplement: SourceData FS4 — is the source file for Fig. S4. [file JCB_202305065_SourceDataFS4.pdf]
